# Evaluation of ASHA programme in Karnataka under the National Rural Health Mission

**DOI:** 10.1186/1753-6561-6-S5-P12

**Published:** 2012-09-28

**Authors:** Prem Mony, Mohan Raju

**Affiliations:** 1 St. Johns National Academy of Health Sciences, Bangalore, India; 2 Karnataka State Health Systems Resource Centre, Bangalore, India

## Introduction

The Accredited Social Health Activist (ASHA) programme of the National Rural Health Mission (NRHM) is considered as being vital to achieving the goal of increasing community participation with the health system, and is one of the key components of NRHM, India’s flagship programme in health launched in 2005. The problem of evaluating ASHA is compounded by multiple and contesting narratives of what constitutes the legitimate role of an ASHA. The discourse on the ASHA’s role centres around three typologies: ASHA as an activist, ASHA as a link worker or facilitator, and ASHA as a community level health care provider. Another problem for evaluation is that the ASHA programme is implemented concurrently with a number of other components of the NRHM such as the *Janani Suraksha Yojana* (JSY) and the emergency transport (108) programme and it is impossible to isolate or attribute outcomes as being due to the ASHA programme alone. Methodologically, there is also no baseline status for comparison after the introduction of the ASHA programme in a classical ‘before-and-after’ study mode. This paper explores the diversity within the ASHA programmes in different districts and overall within Karnataka.

## Methods

The sampling design we adopted was a multi-stage sampling design proposed by the National Health Systems Resources Centre (NHSRC) for all states across India in order to enable comparisons. First step was to choose study districts, then *talukas* within districts, then clusters within *talukas* and then villages within clusters. In Karnataka, three districts were selected. The evaluation methodology included interaction with key informants, in-depth interviews at the state level and a combination of in-depth interviews and focus group discussions with key programme officers at the state, district and sub-district level, ASHAs, auxiliary nurse-midwives (ANM) and *Anganwadi* workers (AWW), members of village health and sanitation committees (VHSC) and beneficiaries.

## Result

ASHA programme was found to be operational in the villages of these three study districts in Karnataka. The ASHA workers perform tasks mostly as link workers and community health workers and to only a small extent as social activists. Within the domain of their link worker role, through their home visits to the households of community members they have contributed to improvements in the basic antenatal care and also in increasing the number of institutional deliveries and immunisation. We also found that there is inadequate coverage of marginalized households within villages and hamlets in rural and peri-urban Karnataka.

## Discussion

Special training of ASHAs should be undertaken since one of the primary objectives of the ASHA programme is to improve social justice. The importance of key equity stratifiers such as age, sex, geography and socioeconomic status for several health outcomes need to be emphasised in both the training modules as well as in routine supervision.

## Competing interests

None declared

## Funding statement

None declared

